# Evidence for similarity in symptoms and mechanism: The extra‐pulmonary symptoms of severe asthma and the polysymptomatic presentation of fibromyalgia

**DOI:** 10.1002/iid3.263

**Published:** 2019-08-23

**Authors:** Michael E. Hyland, Joseph W. Lanario, Yinghui Wei, Rupert C. Jones, Matthew Masoli

**Affiliations:** ^1^ School of Psychology University of Plymouth of Plymouth Plymouth UK; ^2^ University Hospitals Plymouth NHS Trust Plymouth UK; ^3^ School of Computing, Electronics and Mathematics University of Plymouth Plymouth UK; ^4^ Department of Respiratory Medicine, Peninsula School of Medicine and Dentistry University of Plymouth Plymouth UK

**Keywords:** biopsychosocial interaction, complexity, fibromyalgia, functional disorder, network, Severe asthma

## Abstract

**Background:**

Asthma is a disease of the lung and a systemic disease. Functional disorders are associated with multiple systemic abnormalities that have been explained by complexity models. The aim was to test the similarity in type and aetiology between the extra‐pulmonary symptoms of severe asthma and the symptoms of fibromyalgia.

**Methods:**

One Hundred patients recruited from a specialist severe asthma clinic and 1751 people reporting different functional disorder diagnoses recruited via the internet completed the same 60‐item questionnaire. Symptom patterns were compared between groups using a new measure, the symptom pattern similarity index where 0 = no relationship, 1 = identical patterns between groups.

**Results:**

Severe asthma patients report numerous extra‐pulmonary symptoms. The similarity index between the symptom pattern of the asthma patients with other groups was irritable bowel syndrome = 0.54, chronic fatigue syndrome = 0.69, and fibromyalgia = 0.75. The index between fibromyalgia and asthma patients with the most and least frequent extra‐pulmonary symptoms was 0.81 and 0.55 respectively.

**Conclusions:**

Patients with severe asthma have numerous extra‐pulmonary symptoms similar in type and pattern to the symptoms of fibromyalgia. The similarity of the symptom pattern between asthma and fibromyalgia increases as the number of extra‐pulmonary symptoms increases as predicted by network theory and previously shown to be the case with other functional disorders. These findings support the hypothesis that functional disorders and extra‐pulmonary asthma symptoms have a common complexity or network aetiology. Evidence based behavioural interventions for fibromyalgia may be helpful for patients with severe asthma reporting extra‐pulmonary symptoms.

## 1 INTRODUCTION

Asthma is a disease of the lung but also a systemic disease,^1^, ^2^, ^3^ with well‐established comorbidities.^4^, ^5^, ^6^ Although functional disorders are less commonly cited as comorbidities of asthma they include fibromyalgia syndrome (FMS),^7^, ^8^, ^9^ chronic fatigue syndrome (CFS),^9^, ^10^ and irritable bowel syndrome (IBS)^11^). Functional disorders are polysymptomatic and have multiple, systemic biological abnormalities. Although they both involve systemic disturbance, the relation between the symptoms of asthma and functional disorders is unknown. Severe asthma patients have a high level of extra‐pulmonary symptoms, some of which may relate to the side effects of treatment,^12^ so severe asthma patients provide a useful comparison group with functional disorders.

Despite being described as medically unexplained symptoms, several explanations have been proposed for functional disorders. The first type of explanation is that each of the different syndromes is caused by a specific pathophysiology that is yet to be discovered. This specific pathophysiology may be linked to the immune, neurological and endocrine abnormalities that are associated with functional disorders.^13^, ^14^, ^15^, ^16^, ^17^, ^18^, ^19^, ^20^ The second type of explanation is that all the functional disorders are caused by a common psychological mechanism,^21^ such as somatization (hence, somatoform disorders) or some form of cognitive disturbance^22^, ^23^ A third type of explanation, commonly labelled biopsychosocial, combines psychological and biological mechanisms in a single model to explain both the biological and psychological abnormalities of functional disorders. These models take a variety of forms, for example, combining neural and psychology,^24^ neural, gut, and psychology^25^ and neural, immune, endocrine, and psychology.^26^

A common theme in the biopsychosocial models is that the body is an adaptive system, and symptoms are the result of some form of adaptation to situational or behavioural events.^24^, ^26^ Because complex network structures provide the architecture for learning (ie, adaptation) in artificial intelligence systems, it has been suggested that complexity theory may provide insights into the underlying mechanisms.^27^ The adaptive network theory applies network theory to a biological system with biopsychosocial inputs. The theory assumes that biological pathology causing mechanisms are causally connected in a network, which then adapts according to network learning rules.^28^, ^29^ Adaptation results from inputs to the network which include psychological as well as biological inputs, and the resulting biological adaptation is associated with somatic and psychological symptoms.

The symptoms of functional disorders are to some extent shared between different functional disorders.^30^ Psychological models of functional disorders explain the commonality in symptomatology across functional disorders. Biological explanations explain the specificity of symptoms between functional disorders. Biopsychosocial theories explain both the specificity and commonality that is a feature of functional disorders.^30^ The adaptive network theory, however, makes one additional prediction compared to other biopsychosocial theories, namely, that the commonality between functional disorders increases with severity. This prediction stems from the assumption of a network structure. In a network of mutually activating nodes, increase in one part of the network activates increases activity across the whole network, such that differentiation between the original activating site and elsewhere becomes lost as severity increases. This prediction has been confirmed using a machine learning form of analysis with the additional finding that the connection strengths between the outgoing connections also increased with severity, showing that the underlying network structure (and therefore adaptation) varies with severity.^29^

If the polysymptomatic presentation of IBS, FMS, and CFS is due to a causal network of biological, symptom causing mechanisms, then it is plausible that the asthma causing mechanisms as well as systemic effects of treatment could be causally linked to this network to a greater or lesser extent. If the extra‐pulmonary symptoms of asthma have the same network aetiology as IBS, FMS and CFS then the symptomatology should exhibit the same properties.

Two predictions stem from the hypothesis that the functional disorder symptoms and the extra‐pulmonary symptoms of asthma have the same etiology. First, the relative frequency of the different extra‐pulmonary symptoms in a group of patients with severe asthma should be similar to the relative frequency of those symptoms in groups of people diagnosed with IBS, FMS, and CFS. Second, the degree of similarity should be greater in those asthma patients with more extra‐pulmonary symptoms. Previous research shows that as symptom frequency increases, the pattern of symptoms (ie, relative frequency of symptoms) in functional disorder groups become more similar to those with fibromyalgia.^29^ Therefore, the extra‐pulmonary symptoms of people with severe asthma should approximate more to the symptom pattern of fibromyalgia as the number of extra‐pulmonary symptoms increases.

The symptom pattern is defined by the *relative* frequency of one symptom to all others symptoms in a group of people, so the symptom pattern is independent of the absolute frequency of symptoms. For example, if symptom A is twice as common as symptom B in one group of patients, and the same ratio is found in another group, then the two groups have the same symptom pattern even though the absolute frequency of symptoms may be different between the two groups.

This paper has three aims. The first is to compare the type and number of extra‐pulmonary symptoms of severe asthma with the polysymptomatic presentation of IBS, FMS, and CFS. A second aim is to use a quantitative method to assess the degree of similarity between the symptom pattern of severe asthma and those of people with IBS, FMS and CFS to determine which of these three functional disorders is most similar to severe asthma. A third aim is to test the hypothesis that those severe asthma patients with the more extra‐pulmonary symptoms are more similar in symptom pattern to FMS compared with those asthma patients with less extra‐pulmonary symptoms—that is, that the extra‐pulmonary symptoms of asthma exhibit the same property of convergence with severity as do functional disorders and predicted by an underlying network mechanism.

## 2 METHODS

### 2.1 Participants

#### 2.1.1 Asthma sample

Patients aged greater than or equal to 16 years and attending a specialist severe asthma clinic were invited to participate as part of a questionnaire validation study.^31^ All patients were diagnosed with severe asthma as defined by the European Respiratory Society (ERS) and American Thoracic Society (ATS) guidelines^32^ and were excluded if they had another condition that could contribute significantly to their respiratory symptoms, for example, lung cancer, heart failure or COPD.

#### 2.1.2 Functional disorders sample

People over the age of 18 years were recruited through IBS, CFS, and FMS patient self‐help websites as part of another study.^29^

### 2.2 Questionnaire

#### 2.2.1 General symptom questionnaire

The questionnaire for assessing extra‐pulmonary symptoms in asthma was selected so as to be able to provide a comparison with an existing data set obtained from a separate study.^29^ The questionnaire was based on an established general population symptom questionnaire^33^ but with items added that are indicative of functional disorders such as FMS and CFS (See online Appendix 1 for more details). The questionnaire assesses the frequency of somatic and psychological symptoms on a 6‐point Likert scale (the value scoring for each response shown in brackets): “Never or almost never” (1), “Less than 3 or 4 times per year” (2), “Every month or so” (3), “Every week or so” (4), “More than once per week” (5) or “Every day” (6). The *General symptom questionnaire* (*GSQ*) *score* was calculated from the mean of all items. The number of *weekly nonrespiratory symptoms* reported was calculated by counting the number of items with a score of 4 or more. The number of *daily nonrespiratory symptoms* was calculated by counting the number of items with a score of 6. Higher scores indicate more symptoms. There were 60 identical symptoms for which data were obtained from the asthma and functional disorder groups.

### 2.3 Clinic data

The following clinic data were obtained for the asthma sample: spirometry (forced expiratory volume in 1 second [FEV1%]), treatment step as defined by the Global Initiative for Asthma (GINA),^22^ and body mass index (BMI).

### 2.4 Procedure

#### 2.4.1 Asthma sample

After providing written informed consent, participants either completed the questionnaires at home or during their clinic visit.

#### 2.4.2 Functional disorder sample

These data were collected as part of another study.^29^ In an online survey, participants who reported having received a doctor's diagnosis of IBS, CFS or FMS, provided consent and completed an online symptom questionnaire and indicated their age, sex.

Questionnaires from the asthma sample were deemed incomplete if 15 or more items were missing. The online data collection of the functional disorder sample precluded missing items as only complete questionnaires could be submitted.

### 2.5 Ethical approval

Data collection from the asthma sample was approved by the Plymouth Hospitals NHS Trust and REC/HRA, ethical approval number 16/NE/0188, IRAS ID: 207601. All patients provided informed written consent. Ethical approval for data collected in the online study^29^ was provided by the University of Plymouth, Faculty of Health and Human Science Ethics committee. Participants for this study provided informed consent online.

### 2.6 Analysis

Descriptive statistics of symptoms within groups is provided by the percentage of people reporting a symptom at two levels of frequency: at least once per week and once per day.

The symptom pattern of a group is defined by the means of all the symptom scores of that group. A symptom pattern similarity index was developed to compare the similarity of the symptom patterns between groups, where one = identical pattern and zero = unrelated pattern. To calculate this index form, first the mean score for each symptom was calculated for each group (asthma, IBS, FMS, and CFS). Each of these symptom means falls along a scale of 0 to 6, and together these symptoms form the symptom pattern for any group. Two groups were defined as having an identical pattern if the similarity index is 1, in which each of the different symptom means in the two groups are the same after adding a constant to all symptoms in one of the groups (the constant can be zero, in which case not only is the pattern identical but so is severity). To calculate the symptom pattern similarity index between zero and one, Pearson's correlations were calculated where symptoms were treated as cases and groups as variables. We refer to this calculation as a symptom pattern similarity index rather than a correlation coefficient to avoid confusion with the traditional use of correlation. Note that if the correlation were negative this would create a dissimilarity index where − 1 = absolute opposite of pattern. Further details about this index are provided in Appendix 1 (why a Pearson rather than a Spearman and the dissimilarity index).

To examine to what extent the symptom pattern index of one group was a unique contributor to the symptom pattern of another, multiple regressions were carried out using the means of symptoms scores for each of the groups as predictor and dependent variables.

## 3 RESULTS

Participants: Of the 174 patients with severe asthma asked to take part, 20 declined and of the remaining 154 participants, 53 did not return their questionnaires by post, and one participant missed out 20 questions from the GSQ making the data ineligible. Completed questionnaires were received from 100 patients.

Of the 1751 functional disorder participants who completed online questionnaires in a separate study,^18^ 900 people reporting a single functional disorder diagnosis of whom 370 reported IBS, 384 reported FMS, and 146 reported CFS are included for comparison. The remaining 851 had some combination of IBS, FMS, and CFS.

**Figure 1:**
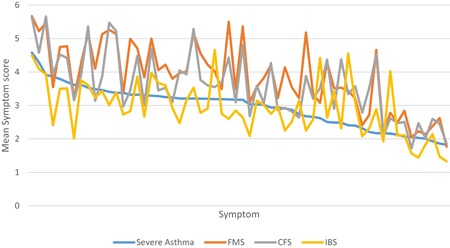
Graphical representation of the relationship between four symptom pattern indices. The line created by the severe asthma group is a monotonic decreasing line because the symptoms along the *x* axis are ordered by magnitude of the symptom mean. The symptom with the greatest mean value is to the left of the *x* axis. The mean symptoms of the IBS group are most similar in magnitude to the severe asthma symptoms. However, the symptom pattern index of the severe asthma group is most similar to the FMS group because the line created by the FMS group is most parallel to the monotonic decrease of the severe asthma group. CFS, chronic fatigue syndrome; FMS, fibromyalgia syndrome; IBS, irritable bowel syndrome

Demographic data and mean questionnaire data of the severe asthma and functional disorder samples (IBS, FMS, and CFS) are shown in Table 1. The frequency of weekly and daily extra‐pulmonary symptoms (ie, GSQ items) for all four groups is shown in Table 2. Table 2 is limited to the 40 most frequent weekly extra‐pulmonary symptoms reported in the asthma sample. The full list of symptoms is shown in the online Appendix 2. Figure 1 provides a graphical representation of the same data, to show how the pattern of IBS, FMS, and CFS groups differs from that of the severe asthma group. The symptom pattern of the severe asthma group forms a decreasing monotonic line because the symptoms have been ordered in terms of their mean values.

**Table 1:** Characteristics of participants

	Severe asthma		IBS		FMS		CFS	
	Mean (SD)	n (%)	Mean (SD)	n (%)	Mean (SD)	n (%)	Mean (SD)	n (%)
Age	52.3 (14.9)	…	50.35 (15.66)	…	52.67 (11.49)		44.24 (14.01)	
Range	17‐79		18‐89		21‐90		17‐76	
Sex								
Female	…	63 (63)		314 (84.9)		362 (94.3)		126 (86.3)
Male		37 (37)		56 (15.1)		22 (5.7)		20 (13.7)
GSQ average score (1‐6)	2.94 (1.06)		2.90 (0.78)		3.81 (0.73)		3.55 (0.70)	
FEV1%	69.72 (18.26)							
Treatment (GINA step)								
Step 4		61 (61)						
Step 5		39 (39)						

Abbreviations: CFS, chronic fatigue syndrome; FEV1, forced expiratory volume in 1 second; FMS, fibromyalgia syndrome; GSQ, General symptom questionnaire; IBS, irritable bowel syndrome.

**Table 2:** Nonrespiratory symptoms limited to the 40 (out of 65) most frequent symptoms occurring weekly in the severe asthma sample

	Weekly symptom				Daily symptom			
Symptoms	Severe asthma	IBS	FMS	CFS	Severe asthma	IBS	FMS	CFS
Waking up still feeling tired, %	73.7	75.4	96.9	95.2	45.5	36.2	78.9	78.1
n	73	279	372	139	45	134	303	114
Waking up often at night, %	69.7	62.4	89.8	74.0	38.4	26.2	58.1	39.0
n	69	231	345	108	38	97	223	57
Fatigue for no reason, %	61.5	62.2	95.3	95.9	27.1	19.7	64.8	78.1
n	59	230	366	140	26	73	249	114
Easily feel too hot/sweating, %	61.0	51.1	79.7	75.3	28.0	20.5	45.6	36.3
n	61	189	306	110	28	76	175	53
Feeling out of breath for no reason, %	58.6	26.5	52.1	62.3	27.3	6.8	20.8	20.5
n	58	98	200	91	27	25	80	30
Difficulty getting to sleep, %	58.0	49.2	79.9	72.6	28.0	14.9	45.8	36.3
n	58	182	307	106	28	55	176	53
Hands tremble or shake, %	57.0	18.1	48.4	46.6	25.0	4.6	11.2	10.3
n	57	67	186	68	25	17	43	15
Irritable, %	55.6	55.4	71.4	63.0	15.2	13.8	21.1	14.4
n	55	205	274	92	15	51	81	21
Difficulty concentrating, %	53.1	51.9	90.4	93.8	14.3	14.6	49.2	54.1
n	52	192	347	137	14	54	189	79
Itchy skin, %	51.5	41.6	66.7	41.8	21.2	14.9	23.7	8.9
n	51	154	256	61	21	55	91	13
Fatigue increasing the day after you are active, %	50.0	40.5	93.0	97.9	28.3	12.7	54.4	61.6
n	46	150	357	143	26	47	209	90
Itchy eyes, %	49.0	36.2	57.8	45.2	13.0	8.6	20.8	8.2
n	49	134	222	66	13	32	80	12
Very cold hands or feet, %	48.5	59.7	79.4	72.6	25.3	30.3	46.9	47.3
n	48	221	305	106	25	112	180	69
Memory problems, %	48.0	48.6	90.4	91.1	20.0	14.3	52.3	58.2
n	48	180	347	133	20	53	201	85
Back pain, %	47.9	45.9	87.5	58.2	24.5	15.1	58.6	28.8
n	45	170	336	85	23	56	225	42
Urinating two or more times per night, %	47.0	32.2	46.1	39.7	27.0	14.1	25.3	17.1
n	47	119	177	58	27	52	97	25
Thirsty all the time, %	47.0	40.0	68.8	52.7	18.0	11.4	33.6	28.8
n	47	148	264	77	18	42	129	42
Easily feel too cold, %	47.0	61.4	84.1	76.0	20.0	31.1	55.7	47.3
n	47	227	323	111	20	115	214	69
Mental fog, %	46.9	50.5	90.9	93.2	15.3	14.3	49.0	56.2
n	46	187	349	136	15	53	188	82
Cramps in leg, foot or bottom, %	46.0	26.2	59.9	39.0	15.0	1.9	19.3	4.8
n	46	97	230	57	15	7	74	7
Feeling anxious for no reason, %	45.5	53.0	64.1	50.0	14.1	20.3	22.1	15.1
n	45	196	246	73	14	75	85	22
Bloating of the stomach, %	45.4	81.1	66.9	53.4	5.2	30.8	17.4	12.3
n	44	300	257	78	5	114	67	18
Sensitive or tender skin, %	44.9	34.6	84.6	51.4	25.5	14.6	52.6	13.7
n	44	128	325	75	25	54	202	20
Pain in legs and arms (which is not due to hard exercise), %	44.3	29.5	94.8	71.9	20.6	10.5	71.4	37.0
n	43	109	364	105	20	39	274	54
Numbness/tingling/pins and needles, %	44.0	31.6	75.3	55.5	11.0	7.0	39.8	17.1
n	44	117	289	81	11	26	153	25
Jittery, easily startled, often worried, %	44	52.7	68.8	51.4	18	19.2	25.8	17.8
n	44	195	264	75	18	71	99	26
Pain increasing the day after you are active, %	43.8	32.2	94.0	85.6	20.8	8.6	59.6	39.0
n	42	119	361	125	20	32	229	57
Racing heart, %	43	34.3	53.6	56.8	17	3.8	11.2	18.5
n	43	127	206	83	17	14	43	27
Restless legs, %	41.4	…	…	…	14.1	…	…	…
n	41	…	…	…	14	…	…	…
Blocked nose, %	40.8	29.5	47.7	41.1	18.4	6.8	15.6	13.0
n	40	109	183	60	18	25	60	19
Very vivid dreams, %	40.4	41.6	51.0	53.4	11.1	7.6	13.8	13.7
n	40	154	196	78	11	28	53	20
Headaches, %	40	38.9	65.9	63.7	11	5.4	16.1	17.8
n	40	144	253	93	11	20	62	26
More clumsy than others, %	39.8	32.7	69.0	69.9	17.3	9.5	26.8	28.8
n	39	121	265	102	17	35	103	42
Swollen painful joints	39.4	22.7	66.1	39.7	18.2	8.9	38.0	10.3
n	39	84	254	58	18	33	146	15
Swollen painful joints, %	39.4	0.0	0.0	0.0	18.2	0.0	0.0	0.0
n	39	0	0	0	18	0	0	0
Chest pain, %	36.7	19.5	42.7	30.1	9.2	2.7	8.9	5.5
n	36	72	164	44	9	10	34	8
Depression, %	36.4	34.3	53.9	38.4	17.2	13.8	26.8	11.0
n	36	127	207	56	17	51	103	16
Running nose, %	36.4	33.2	41.9	32.9	9.1	9.5	11.5	9.6
n	36	123	161	48	9	35	44	14
Face flushes, %	35.7	27.8	53.9	36.3	15.3	7.6	15.6	8.2
n	35	103	207	53	15	28	60	12
Pain moving from one place of body to another on different days, %	35.4	24.1	89.1	62.3	16.7	7.3	58.6	25.3
n	34	89	342	91	16	27	225	37

*Note*: The percentage and (number) of participants experiencing each symptom in four groups: severe asthma (n = 100), IBS (n = 370), FMS (n = 382), and CFS (n = 146).

Abbreviations: CFS, chronic fatigue syndrome; FMS, fibromyalgia syndrome; IBS, irritable bowel syndrome.

The median number of daily extra‐pulmonary symptoms reported by the severe asthma sample was 6 and that of the weekly extra‐pulmonary symptoms was 21. Four asthma patients reported zero weekly nonrespiratory symptoms and 19 reported zero daily nonrespiratory symptoms.

The means of each symptom in the different groups is shown in Appendix 2. The symptom pattern similarity index (ie, the metric of how similar the pattern of symptomatology is between groups) is shown in Table 3. The results show that the symptom pattern of severe asthma is most similar to FMS (the index = 0.75) followed by CFS (0.66) followed by IBS (0.57). The highest symptom pattern similarity index is between FMS and CFS (0.88).

**Table 3:** Pattern similarity indexes between groups of people

	Asthma	IBS	FMS	Asthma‐low	Asthma‐high
Asthma				0.90	0.96
IBS	0.57			0.60	0.49
FMS	0.75		0.57	0.55	0.81
CFS	0.66	0.56	0.88	0.49	0.72
Asthma‐low					0.75

Abbreviations: CFS, chronic fatigue syndrome; FMS, fibromyalgia syndrome; IBS, irritable bowel syndrome.

**Figure 2:**
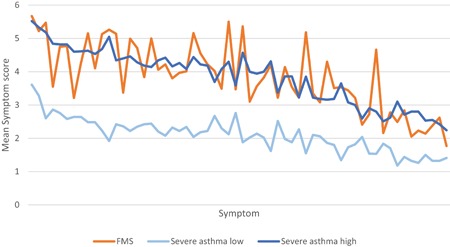
Graphical relationship of the relationship between three symptom pattern indices. The symptoms along the *x* axis are in the same order as for Figure 1 so that the two asthma groups would average to the monotonic line shown in Figure 1 FMS, fibromyalgia syndrome

To test whether the symptom pattern similarity index varies with severity, the severe asthma sample was dichotomised into two groups based on the patients’ mean GSQ scores: Those below the median GSQ score (severe asthma‐low) and those above the median GSQ score (severe asthma‐high), there being 50 patients in either group. The mean symptom scores for these two asthma groups is shown in Appendix 2, a graphical representation is shown in Figure 2 and the correlations in Table 3.

Two multivariable linear regressions were carried out to assess whether the symptom patterns of FMS and IBS (ie, the mean symptom scores of the FMS and IBS groups) were predictors of (a) the symptom pattern of the severe asthma‐high group and (b) the symptom pattern of the severe asthma‐low group (ie, the mean symptom scores of the dichotomised asthma groups). In these multi‐variable regressions, only the pattern of FMS (*P* < .001, *β* = .79) but not the pattern of IBS (*P* = .71, *β *= 0.37) predicted the pattern of severe asthma‐high. Both the pattern of FMS (*P* = .014, *β* = .31) and the pattern of IBS (*P* = .001, *β* = .42) predicted the pattern of severe asthma‐low.

## 4 DISCUSSION

The result of this survey shows that the majority of patients attending a severe asthma clinic report many different extra‐pulmonary symptoms. The number of different extra‐pulmonary symptoms reported varies between patients with a median of 21 symptoms per week and only a small minority reporting none. Inspection of the type of symptoms reported shows that they are similar to the polysymptomatic presentation of patients with IBS, and CFS, but particularly FMS. For example, 35% of the asthma patients reported the symptom of “pain moving from one part of the body to another on different days” at least once per week. This symptom, reported by 89% of the FMS patients is indicative of central sensitivity syndrome^13^ rather than damage. Cognitive disturbance of various kinds were reported by about 50% of the asthma patients, and although this symptom is less common than that of the FMS patients (where it is about 90%) it is nevertheless a symptom often referred to as ‘fibro‐fog’^34^ by FMS patients but also common in CFS patients. Stomach pain was reported by 20% of the asthma patients weekly, compared with 79% for IBS. The levels of depression (36%) and anxiety (45%) are comparable with those of an international survey of severe asthma.^35^

Similarity in symptoms between two groups does not mean similarity of cause. Irritability is a symptom of all four groups but is also a side effect of oral corticosteroids. To examine evidence for similarity of cause, we developed a metric, the symptom pattern similarity index, varying between 0 and 1 to express the extent to which the pattern of symptoms is similar between groups, independent of the overall frequency of symptoms (1 = the patterns are identical; 0 = patterns are unrelated). Using this metric, the symptom pattern similarity index of the severe asthma sample is most similar to the FMS group (0.75) and least similar to the IBS group (0.57). Figure 1 provides a graphical representation of the difference between symptom similarity and symptom pattern similarity. Of the three groups, the mean symptoms of the IBS are most similar to the severe asthma group. However, the symptom pattern index, is defined by the relationship between the means of symptoms within a group, not by the means themselves. The line in Figure 1 formed by the FMS group is almost entirely above that of the severe asthma, but because it approximates more to a line parallel to the severe asthma group, the correlation between the FMS and the severe asthma group is greater than that between the IBS and severe asthma group.

Network theory suggests that similarity between groups should increase with severity because increased severity is associated with increased pathology over the whole network. Therefore symptom patterns within different diagnostic groups should converge as severity increases, consistent with evidence elsewhere that people diagnosed with IBS or CFS become more similar to the more severe group of FMS as the frequency of IBS and CFS symptoms increases.^29^ We found that people reporting a diagnosis of FMS report the most frequent symptoms compared with IBS and CFS (see Table 1). We found that those asthma patients who had more extra‐pulmonary symptoms were more strongly related to the FMS group using our index (0.81) compared to those with less extra‐pulmonary symptoms (0.55). Figure 2 provides a graphical representation of these data. The severe asthma high and severe asthma low lines are approximate mirror images of each other as the mean values of the high and low groups are the same as the means for the total group—and therefore the decreasing line shown in Figure 1. However, in this case, the mean values of the severe asthma‐high group are similar to the mean values of the FMS group, and the peaks and troughs of the severe asthma‐high line tend to mirror the peaks and troughs of the FMS line, showing that the FMS and severe asthma‐high groups tend to be parallel, though in this case not monotonic. By contrast, the peaks and troughs of the severe asthma‐low line tends to oppose the peaks and troughs of the FMS lines. As the correlation shows in Table 3, the pattern index of the severe asthma‐high group is more similar to the FMS group than it is to the severe asthma‐low group.

This correlation coefficients and graphs show that not only are the extra‐pulmonary symptoms of asthma similar (though for most symptoms less frequent) than those reported by FMS, but also that the extra‐pulmonary symptoms exhibit the same property of convergence with severity as predicted by network theory. The property of convergence with the symptom pattern FMS can be explained by a biopsychosocial model *only* if it includes a network architecture. Models without a network architecture explain symptoms by a combination of biological and psychological mechanisms. In any population, different symptoms have different frequencies such that there is a symptom pattern that characterises for that population. If symptoms have a psychological cause, then, irrespective of whether one or several psychological mechanisms are involved, increase in the severity of one or more psychological mechanisms should increase all symptom reporting by the same proportional amount. The consequence is that as the severity of the psychological cause increases, the symptom pattern for severe and less severe groups should be the same. In the case of biological causes, increased pathology of a biological cause should increase only the symptoms associated with that biological cause. As our evidence shows that increase in symptom frequency (including somatic symptoms) is associated with a convergence towards the FMS symptom pattern, our data are inconsistent with nonnetwork explanations, but consistent with a network of biological mechanisms.

The symptom pattern similarity index between CFS and FMS is high (0.87), consistent with clinical observation that the symptoms of these two groups are similar despite a different diagnostic procedure. However, this similarity of pattern makes differentiation between CFS and FMS unreliable. The relationship between FMS and IBS is lower (0.57), but still moderately strong. We wished to determine whether the pattern of IBS explained additional variation of the asthma pattern compared to FMS. In the case of asthma patients with more extra‐pulmonary symptoms, only FMS but not IBS was a significant independent predictor of the asthma pattern, that is, any similarity in pattern between IBS and asthma can be entirely explained by the similarity between FMS and IBS. In the case of asthma patients with less extra‐pulmonary symptoms, both FMS and IBS were independent predictors of the asthma pattern. We cannot say whether the latter effect occurs because some of the asthma patients exhibit the IBS and some the FMS pattern, or whether there is a tendency for both patterns to manifest in the same patient, but the results are consistent with the network theory. For patients with less severe pathology in the network, there is greater opportunity for activation at particular nodes in the network (ie, greater opportunity for localised pathology), and so there is less convergence between symptom patterns between groups when severity is low. These findings are also consistent with a network but not other explanations, including nonnetwork biopsychosocial explanations.

### 4.1 Limitations

All groups are convenience samples. Group membership of the functional disorder groups is based on clinical criteria that may differ from diagnostic criteria. The severe asthma sample was recruited from one centre in the UK. The prevalence of FMS within the asthma group could not be determined because of underdiagnosis of FMS in clinical practice exacerbated by significant differences between different diagnostic criteria.^36^, ^37^ Statistical bias can arise when correlations are calculated from split groups. There is no statistic available for testing whether differences in the symptom pattern index are significant or not. The reason is explained in the Appendix.

### 4.2 Conclusions and clinical relevance

Our results support the hypothesis that the extra‐pulmonary symptoms of asthma have a similar aetiology to FMS, and that this aetiology is based on a causal network of symptom causing mechanisms to which the mechanisms of asthma and its treatment are causally linked. This conclusion is supported by existing data showing that asthma, FMS and CFS share several nondiagnostic biological abnormalities, including raised systemic inflammation,^38^, ^39^, ^40^, ^41^ hypothalamic‐pituitary‐adrenal axis dysregulation^42^, ^43^, ^44^ and low magnesium levels.^45^, ^46^ Evidence that fibromyalgia is a risk factor for asthma exacerbations^7^ as are nonasthma related visits to the GP,^47^ is consistent with an inflammatory causal pathway between systemic inflammation and inflammation in the lung.

There are three possible applications of network theory to the management of severe asthma. First, if variation in systemic inflammation interacts with inflammation in the lung, then it is plausible that evidence based interventions for FMS will have a beneficial effect in asthma, but only for those patients with high levels of extra‐pulmonary symptoms. Studies on behavioural interventions for severe asthma are needed, and such interventions may also act to prime pharmacological treatments. Second, as asthma treatments affect the immune system in different ways, it is plausible that systemic corticosteroids and biologic treatments have radically different effects on extra‐pulmonary symptoms and therefore quality of life. Quality of life scales currently used in severe asthma studies have not been optimised for this patient population,^48^ and may therefore underestimate the benefit of newer, targeted forms of treatment. A recently developed quality of life scale for severe asthma,^31^ and the inclusion of measures of extra‐pulmonary symptoms will provide a more valid representation of quality of life and symptoms. Third, patients’ reports of the benefit of biologic treatment varies. As these treatments will interact with the existing state of a network of biological mechanisms, it is possible that extra‐pulmonary symptom patterns predict benefit pharmacological treatments where the outcome varies. Predictor studies for biologic treatments using symptom patterns may help provide a more personalised use of new treatments.

## DATA ACCESSIBILITY

Both data sets (asthma and functional disorders) are available as separate files (Excel or SPSS format) from the corresponding author.

## Supporting information

Supporting information
