# Supplementary material for: Evidence for similarity in symptoms and mechanism: The extra‐pulmonary symptoms of severe asthma and the polysymptomatic presentation of fibromyalgia
Source: Immun Inflamm Dis. 2019 Aug 23;7(4):239–49. doi: 10.1002/iid3.263 (PMC6842811; doi:10.1002/iid3.263)
Supplement: Supplementary file 1 — Supporting information [file IID3-7-239-s001.docx]

**Appendix 1**

*Development of the General Symptom Questionnaire*

The general symptom questionnaire (GSQ) was developed as a clinical tool to use as part of a psycho-educational intervention for fibromyalgia syndrome (FMS) ([www.bodyreprogramming.org](http://www.bodyreprogramming.org) ). The questionnaire is used in the first session to introduce patients to the idea that FMS is not only a condition of pain but includes other symptoms as well. A 61 item questionnaire was produced by taking the items from a well-established general population symptom questionnaire [12], and adding and modifying symptoms on the basis of known symptoms and on the basis of feedback from patients on earlier versions. That 61 item questionnaire was then used in an internet survey of IBS, FMS and CFS [17] where participants were also able to write down any symptom not present in the questionnaire. One of the 61 items was then dropped as the frequency of occurrence was deemed too low, and five items were added, these being items (or synonyms) that were frequently added as additional items. The resulting 65 item questionnaire is now used in clinical practice for treating fibromyalgia (see [www.bodyreprogramming.org](http://www.bodyreprogramming.org)) and is the one that was used in this study. Table 1S shows the data from the internet study using the 61 item questionnaire [17], as well as the items from the 65 item questionnaire used in this study. A validation paper for the questionnaire is currently in press (Hyland ME, Bacon A, Lanario JW, Davies A. Symptom frequency and development of a generic functional disorder symptom scale suitable for use in studies with IBS, FMS or CFS patients. Chronic Disease and Translational Medicine, in press.)

*The pattern similarity index: measurement principles*

Any measure of pattern similarity is based on assumptions. Our approach is based on the assumption that a pattern of symptoms can be defined by the means of each of the individual symptoms within groups. The symptom means vary between 0 and 6, and together form a pattern. Two groups are defined as having an identical symptom pattern if each of the different symptom means in the two groups are same after adding a constant to all symptoms in one of the groups. The assumption is based on the principle that a pattern can vary independently of severity such that the same pattern can occur at different levels of severity. It is clear that symptom patterns are the same if the symptom means are identical between groups. However, we wished to classify symptom patterns as identical where all the means scores of one group is shifted up or down by a constant amount on the 1 – 6 scale.

Pearson correlations assume that the 1 – 6 scale on which the means fall is a ratio or equal interval scale. If the 1 – 6 scale is not an equal interval scale, then there can be some bias, but the bias will be small except where there are substantial differences in severity. In the case of the GSQ, the response format is such that any deviation from a ratio scale is likely to be very small. One possible response to the possibility of a non-ratio scale would be to use a Spearman correlation However, there are problems associated with this strategy. If a Spearman correlation is used then symptom pattern score will have a value of one both where (a) the means symptom scores are identical between groups and (b) also where they mean symptom scores differ by a variable amount even though the order of magnitude between symptoms remains the same.

The insensitivity of the Spearman under certain circumstances, can be illustrated with the following exercise. Consider two hypothetical groups with three symptoms. In one group, the symptoms have values 1, 2, 3 and in the other group 2, 3, 4 respectively. For both the Pearson and the Spearman correlation, the coefficient is 1.0. Now consider two groups with values 1, 2, 6, and 2, 3, 4. The Spearman coefficient remains 1.0, but the Pearson is 0.945. The Spearman has failed to differentiate between the identical pattern, and one where there is a difference in pattern, as defined by the rule given at the start of this section. It is because the Spearman correlation is insensitive to what may be important absolute differences in magnitude of means along the 1 – 6 scale that we have selected the Pearson correlation as being the more suitable for the pattern similarity index.

The symptom pattern similarity index is a new index and there is no statistical test currently available. The most interesting test would be the significance between different indices. This comparison cannot be made using the usual test of difference between r-values. The reason is that the values in the correlation matrix do not come from individual people but from groups of people, such that the more people in the group, the more reliable the symptom pattern becomes. Additionally, the less variability within each item within each group, the more reliable the symptom pattern becomes. So, for comparison between symptom patterns, statistical significance is affected by three variables: the number of symptoms (i.e., the degrees of freedom when correlations are used in their normal way), the number of people in the two groups and the variability of items. In our study, the smallest group size is for the high and low severe asthma groups – i.e., 50 people. This number is likely to produce a fairly stable symptom pattern index, and groups with 100 or more people will be even more stable. Hence, small differences in correlations between the symptom patterns is likely to be significant – and much more so than a comparison of a correlation with 59 degrees of freedom - but we cannot provide a figure.

The similarity index varies between 1 and 0. However, it is also possible for the correlations to be negative and vary between -1 and 0, and these would constitute a dissimilarity index. For example, if the mean symptom scores were 1, 2 , 3 in one group and 3, 2, 1 in the other, then the correlation would be -1 indicating that the pattern of the two groups are polar opposites –i.e., they cannot be more different. Network theory suggests that there should be only similarity indexes, but there may be theoretical mechanisms leading to a dissimilarity index. Although this paper focuses only on similarity, dissimilarity provides a possible mechanism for differential diagnosis.

Appendix 2

*Table 1S*. Symptoms occurring weekly or more, or daily in four groups of participants. The **percentage** and (number) of participants experiencing each symptom in four groups: severe asthma (n = 100), IBS (n= 370), FMS (n = 382) and CFS (n = 146).

|  | Weekly Symptom | | | |  | Daily Symptom | | | |
| --- | --- | --- | --- | --- | --- | --- | --- | --- | --- |
| Symptoms | Severe Asthma | IBS | FMS | CFS |  | Severe Asthma | IBS | FMS | CFS |
| Waking up still feeling tired | **73.7** | **75.4** | **96.9** | **95.2** |  | **45.5** | **36.2** | **78.9** | **78.1** |
|  | *73* | *279* | *372* | *139* |  | *45* | *134* | *303* | *114* |
| Waking up often at night | **69.7** | **62.4** | **89.8** | **74.0** |  | **38.4** | **26.2** | **58.1** | **39.0** |
|  | *69* | *231* | *345* | *108* |  | *38* | *97* | *223* | *57* |
| Fatigue for no reason | **61.5** | **62.2** | **95.3** | **95.9** |  | **27.1** | **19.7** | **64.8** | **78.1** |
|  | *59* | *230* | *366* | *140* |  | *26* | *73* | *249* | *114* |
| Easily feel too hot/sweating | **61.0** | **51.1** | **79.7** | **75.3** |  | **28.0** | **20.5** | **45.6** | **36.3** |
|  | *61* | *189* | *306* | *110* |  | *28* | *76* | *175* | *53* |
| Feeling out of breath for no reason | **58.6** | **26.5** | **52.1** | **62.3** |  | **27.3** | **6.8** | **20.8** | **20.5** |
|  | *58* | *98* | *200* | *91* |  | *27* | *25* | *80* | *30* |
| Difficulty getting to sleep | **58.0** | **49.2** | **79.9** | **72.6** |  | **28.0** | **14.9** | **45.8** | **36.3** |
|  | *58* | *182* | *307* | *106* |  | *28* | *55* | *176* | *53* |
| Hands tremble or shake | **57.0** | **18.1** | **48.4** | **46.6** |  | **25.0** | **4.6** | **11.2** | **10.3** |
|  | *57* | *67* | *186* | *68* |  | *25* | *17* | *43* | *15* |
| Irritable | **55.6** | **55.4** | **71.4** | **63.0** |  | **15.2** | **13.8** | **21.1** | **14.4** |
|  | *55* | *205* | *274* | *92* |  | *15* | *51* | *81* | *21* |
| Difficulty concentrating | **53.1** | **51.9** | **90.4** | **93.8** |  | **14.3** | **14.6** | **49.2** | **54.1** |
|  | *52* | *192* | *347* | *137* |  | *14* | *54* | *189* | *79* |
| Itchy skin | **51.5** | **41.6** | **66.7** | **41.8** |  | **21.2** | **14.9** | **23.7** | **8.9** |
|  | *51* | *154* | *256* | *61* |  | *21* | *55* | *91* | *13* |
| Fatigue increasing the day after you are active | **50.0** | **40.5** | **93.0** | **97.9** |  | **28.3** | **12.7** | **54.4** | **61.6** |
|  | *46* | *150* | *357* | *143* |  | *26* | *47* | *209* | *90* |
| Itchy eyes | **49.0** | **36.2** | **57.8** | **45.2** |  | **13.0** | **8.6** | **20.8** | **8.2** |
|  | *49* | *134* | *222* | *66* |  | *13* | *32* | *80* | *12* |
| Very cold hands or feet | **48.5** | **59.7** | **79.4** | **72.6** |  | **25.3** | **30.3** | **46.9** | **47.3** |
|  | *48* | *221* | *305* | *106* |  | *25* | *112* | *180* | *69* |
| Memory problems | **48.0** | **48.6** | **90.4** | **91.1** |  | **20.0** | **14.3** | **52.3** | **58.2** |
|  | *48* | *180* | *347* | *133* |  | *20* | *53* | *201* | *85* |
| Back pain | **47.9** | **45.9** | **87.5** | **58.2** |  | **24.5** | **15.1** | **58.6** | **28.8** |
|  | *45* | *170* | *336* | *85* |  | *23* | *56* | *225* | *42* |
| Urinating two or more times per night | **47.0** | **32.2** | **46.1** | **39.7** |  | **27.0** | **14.1** | **25.3** | **17.1** |
|  | *47* | *119* | *177* | *58* |  | *27* | *52* | *97* | *25* |
| Thirsty all the time | **47.0** | **40.0** | **68.8** | **52.7** |  | **18.0** | **11.4** | **33.6** | **28.8** |
|  | *47* | *148* | *264* | *77* |  | *18* | *42* | *129* | *42* |
| Easily feel too cold | **47.0** | **61.4** | **84.1** | **76.0** |  | **20.0** | **31.1** | **55.7** | **47.3** |
|  | *47* | *227* | *323* | *111* |  | *20* | *115* | *214* | *69* |
| Mental fog | **46.9** | **50.5** | **90.9** | **93.2** |  | **15.3** | **14.3** | **49.0** | **56.2** |
|  | *46* | *187* | *349* | *136* |  | *15* | *53* | *188* | *82* |
| Cramps in leg, foot or bottom | **46.0** | **26.2** | **59.9** | **39.0** |  | **15.0** | **1.9** | **19.3** | **4.8** |
|  | *46* | *97* | *230* | *57* |  | *15* | *7* | *74* | *7* |
| Feeling anxious for no reason | **45.5** | **53.0** | **64.1** | **50.0** |  | **14.1** | **20.3** | **22.1** | **15.1** |
|  | *45* | *196* | *246* | *73* |  | *14* | *75* | *85* | *22* |
| Bloating of the stomach | **45.4** | **81.1** | **66.9** | **53.4** |  | **5.2** | **30.8** | **17.4** | **12.3** |
|  | *44* | *300* | *257* | *78* |  | *5* | *114* | *67* | *18* |
| Sensitive or tender skin | **44.9** | **34.6** | **84.6** | **51.4** |  | **25.5** | **14.6** | **52.6** | **13.7** |
|  | *44* | *128* | *325* | *75* |  | *25* | *54* | *202* | *20* |
| Pain in legs and arms (which is not due to hard exercise) | **44.3** | **29.5** | **94.8** | **71.9** |  | **20.6** | **10.5** | **71.4** | **37.0** |
|  | *43* | *109* | *364* | *105* |  | *20* | *39* | *274* | *54* |
| Numbness/ tingling/ pins and needles | **44.0** | **31.6** | **75.3** | **55.5** |  | **11.0** | **7.0** | **39.8** | **17.1** |
|  | *44* | *117* | *289* | *81* |  | *11* | *26* | *153* | *25* |
| Jittery. easily startled, often worried | **44** | **52.7** | **68.8** | **51.4** |  | **18** | **19.2** | **25.8** | **17.8** |
|  | *44* | *195* | *264* | *75* |  | *18* | *71* | *99* | *26* |
| Pain increasing the day after you are active | **43.8** | **32.2** | **94.0** | **85.6** |  | **20.8** | **8.6** | **59.6** | **39.0** |
|  | *42* | *119* | *361* | *125* |  | *20* | *32* | *229* | *57* |
| Racing heart | **43** | **34.3** | **53.6** | **56.8** |  | **17** | **3.8** | **11.2** | **18.5** |
|  | *43* | *127* | *206* | *83* |  | *17* | *14* | *43* | *27* |
| Restless legs | **41.4** |  |  |  |  | **14.1** |  |  |  |
|  | *41* |  |  |  |  | *14* |  |  |  |
| Blocked nose | **40.8** | **29.5** | **47.7** | **41.1** |  | **18.4** | **6.8** | **15.6** | **13.0** |
|  | *40* | *109* | *183* | *60* |  | *18* | *25* | *60* | *19* |
| Very vivid dreams | **40.4** | **41.6** | **51.0** | **53.4** |  | **11.1** | **7.6** | **13.8** | **13.7** |
|  | *40* | *154* | *196* | *78* |  | *11* | *28* | *53* | *20* |
| Headaches | **40** | **38.9** | **65.9** | **63.7** |  | **11** | **5.4** | **16.1** | **17.8** |
|  | *40* | *144* | *253* | *93* |  | *11* | *20* | *62* | *26* |
| More clumsy than others | **39.8** | **32.7** | **69.0** | **69.9** |  | **17.3** | **9.5** | **26.8** | **28.8** |
|  | *39* | *121* | *265* | *102* |  | *17* | *35* | *103* | *42* |
| Swollen painful joints | **39.4** | **22.7** | **66.1** | **39.7** |  | **18.2** | **8.9** | **38.0** | **10.3** |
|  | *39* | *84* | *254* | *58* |  | *18* | *33* | *146* | *15* |
| Swollen painful joints | **39.4** | **0.0** | **0.0** | **0.0** |  | **18.2** | **0.0** | **0.0** | **0.0** |
|  | *39* | *0* | *0* | *0* |  | *18* | *0* | *0* | *0* |
| Chest pain | **36.7** | **19.5** | **42.7** | **30.1** |  | **9.2** | **2.7** | **8.9** | **5.5** |
|  | *36* | *72* | *164* | *44* |  | *9* | *10* | *34* | *8* |
| Depression | **36.4** | **34.3** | **53.9** | **38.4** |  | **17.2** | **13.8** | **26.8** | **11.0** |
|  | *36* | *127* | *207* | *56* |  | *17* | *51* | *103* | *16* |
| Running nose | **36.4** | **33.2** | **41.9** | **32.9** |  | **9.1** | **9.5** | **11.5** | **9.6** |
|  | *36* | *123* | *161* | *48* |  | *9* | *35* | *44* | *14* |
| Face flushes | **35.7** | **27.8** | **53.9** | **36.3** |  | **15.3** | **7.6** | **15.6** | **8.2** |
|  | *35* | *103* | *207* | *53* |  | *15* | *28* | *60* | *12* |
| Pain moving from one place of body to another on different days | **35.4** | **24.1** | **89.1** | **62.3** |  | **16.7** | **7.3** | **58.6** | **25.3** |
|  | *34* | *89* | *342* | *91* |  | *16* | *27* | *225* | *37* |
| Fatigue increasing after a cold or sore throat | **35.1** | **19.2** | **53.6** | **57.5** |  | **20.6** | **9.2** | **35.9** | **41.8** |
|  | *34* | *71* | *206* | *84* |  | *20* | *34* | *138* | *61* |
| Ringing in ears | **34.0** | **29.2** | **46.9** | **45.2** |  | **18.0** | **13.2** | **23.4** | **17.8** |
|  | *34* | *108* | *180* | *66* |  | *18* | *49* | *90* | *26* |
| Dizziness or loss of balance | **33.0** |  |  |  |  | **10.0** |  |  |  |
|  | *33* |  |  |  |  | *10* |  |  |  |
| Heartburn | **32.3** | **41.4** | **45.8** | **26.7** |  | **4.2** | **5.7** | **10.4** | **6.8** |
|  | *31* | *153* | *176* | *39* |  | *4* | *21* | *40* | *10* |
| Intolerant to some food | **32.0** | **72.2** | **39.1** | **51.4** |  | **14.0** | **38.9** | **15.9** | **26.7** |
|  | *32* | *267* | *150* | *75* |  | *14* | *144* | *61* | *39* |
| Problems urinating e.g., frequency, hesitancy or pain | **29.6** |  |  |  |  | **18.4** |  |  |  |
|  | *29* |  |  |  |  | *18* |  |  |  |
| Constipation | **29.3** | **50.3** | **51.3** | **32.2** |  | **4.0** | **8.4** | **8.9** | **6.2** |
|  | *29* | *186* | *197* | *47* |  | *4* | *31* | *34* | *9* |
| Feeling very ill for no reason | **29.3** |  |  |  |  | **10.1** |  |  |  |
|  | *29* |  |  |  |  | *10* |  |  |  |
| Blurred vision | **26.5** |  |  |  |  | **13.3** |  |  |  |
|  | *26* |  |  |  |  | *13* |  |  |  |
| Sensitivity to bright lights | **26.0** | **30.5** | **71.4** | **71.9** |  | **14.0** | **10.8** | **34.4** | **34.2** |
|  | *26* | *113* | *274* | *105* |  | *14* | *40* | *132* | *50* |
| Nausea for no reason | **25.3** | **40.0** | **43.8** | **54.1** |  | **6.1** | **5.9** | **7.8** | **9.6** |
|  | *25* | *148* | *168* | *79* |  | *6* | *22* | *30* | *14* |
| Double vision | **21.0** | **6.5** | **25.0** | **27.4** |  | **7.0** | **2.4** | **5.5** | **8.2** |
|  | *21* | *24* | *96* | *40* |  | *7* | *9* | *21* | *12* |
| Choking sensations | **21.0** | **9.5** | **20.6** | **13.0** |  | **4.0** | **1.6** | **2.6** | **2.1** |
|  | *21* | *35* | *79* | *19* |  | *4* | *6* | *10* | *3* |
| Stomach pain | **20.4** | **78.6** | **52.3** | **42.5** |  | **2.0** | **27.0** | **10.4** | **8.9** |
|  | *20* | *291* | *201* | *62* |  | *2* | *100* | *40* | *13* |
| Sensitivity to noise | **19.2** | **36.5** | **78.6** | **76.0** |  | **8.1** | **13.2** | **42.2** | **36.3** |
|  | *19* | *135* | *302* | *111* |  | *8* | *49* | *162* | *53* |
| Feeling faint | **19.2** | **18.9** | **28.6** | **50.7** |  | **2.0** | **2.2** | **5.5** | **11.6** |
|  | *19* | *70* | *110* | *74* |  | *2* | *8* | *21* | *17* |
| Hair Loss | **18.2** |  |  |  |  | **7.1** |  |  |  |
|  | *18* |  |  |  |  | *7* |  |  |  |
| Skin rash | **17.7** | **14.1** | **17.4** | **17.8** |  | **5.2** | **6.5** | **4.7** | **6.2** |
|  | *17* | *52* | *67* | *26* |  | *5* | *24* | *18* | *9* |
| Nightmares/night terrors | **17.2** | **16.5** | **25.0** | **24.7** |  | **7.1** | **2.7** | **2.9** | **3.4** |
|  | *17* | *61* | *96* | *36* |  | *7* | *10* | *11* | *5* |
| Diarrhoea | **17.0** | **67.6** | **32.6** | **28.1** |  | **0.0** | **11.9** | **1.8** | **2.1** |
|  | *17* | *250* | *125* | *41* |  | *0* | *44* | *7* | *3* |
| Twitching of eyelid | **16.2** | **10.8** | **31.5** | **24.7** |  | **2.0** | **0.8** | **3.9** | **1.4** |
|  | *16* | *40* | *121* | *36* |  | *2* | *3* | *15* | *2* |
| Boils or pimples on face or body | **15.2** | **16.5** | **22.7** | **28.8** |  | **5.1** | **6.8** | **7.8** | **8.9** |
|  | *15* | *61* | *87* | *42* |  | *5* | *25* | *30* | *13* |
| Twitching other than eyelid | **14.3** | **5.7** | **32.3** | **28.1** |  | **5.1** | **1.1** | **8.1** | **4.8** |
|  | *14* | *21* | *124* | *41* |  | *5* | *4* | *31* | *7* |
| Loss of voice | **13.1** | **3.5** | **11.7** | **13.0** |  | **3.0** | **0.0** | **1.0** | **2.1** |
|  | *13* | *13* | *45* | *19* |  | *3* | *0* | *4* | *3* |
| Mouth ulcers (sores in mouth) | **13.1** | **6.2** | **15.1** | **14.4** |  | **3.0** | **0.5** | **1.3** | **2.1** |
|  | *13* | *23* | *58* | *21* |  | *3* | *2* | *5* | *3* |
| Head cold, sore throat or 'flu | **6.1** | **5.7** | **14.1** | **29.5** |  | **1.0** | **1.1** | **2.6** | **4.1** |
|  | *6* | *21* | *54* | *43* |  | *1* | *4* | *10* | *6* |
| Cold sores on or near lips |  | **1.9** | **4.4** | **2.1** |  |  | **0.0** | **0.5** | **0.0** |
|  |  | *7* | *17* | *3* |  |  | *0* | *2* | *0* |

*Table 2s*. Mean scores per item per group from which correlations are calculated to create the metric of similarity between symptomatology pattern.

| Symptom | Severe Asthma | FMS | CFS | IBS | Severe asthma low | Severe asthma high |
| --- | --- | --- | --- | --- | --- | --- |
| Waking up still feeling tired | 4.58 | 5.67 | 5.63 | 4.50 | 3.61 | 5.52 |
| Waking up often at night | 4.29 | 5.22 | 4.58 | 4.09 | 3.28 | 5.33 |
| Fatigue for no reason | 3.92 | 5.47 | 5.66 | 3.93 | 2.60 | 5.18 |
| Feeling out of breath for no reason | 3.86 | 3.55 | 3.93 | 2.41 | 2.86 | 4.84 |
| Easily feel too hot/sweating | 3.79 | 4.75 | 4.52 | 3.50 | 2.76 | 4.82 |
| Difficulty getting to sleep | 3.70 | 4.77 | 4.42 | 3.51 | 2.58 | 4.82 |
| Hands tremble or shake | 3.62 | 3.21 | 3.16 | 2.02 | 2.64 | 4.60 |
| Irritable | 3.62 | 4.27 | 3.91 | 3.75 | 2.64 | 4.61 |
| Difficulty concentrating | 3.53 | 5.15 | 5.36 | 3.61 | 2.48 | 4.63 |
| Itchy skin | 3.49 | 4.10 | 3.14 | 3.24 | 2.48 | 4.53 |
| Back pain | 3.46 | 5.13 | 3.86 | 3.43 | 2.23 | 4.68 |
| Fatigue increasing the day after you are active | 3.41 | 5.26 | 5.47 | 3.01 | 1.92 | 5.05 |
| Memory problems | 3.38 | 5.14 | 5.22 | 3.39 | 2.42 | 4.34 |
| Urinating two or more times per night | 3.38 | 3.37 | 2.97 | 2.73 | 2.36 | 4.40 |
| Sensitive or tender skin | 3.32 | 4.99 | 3.45 | 2.83 | 2.22 | 4.46 |
| Very cold hands or feet | 3.32 | 4.71 | 4.48 | 3.86 | 2.35 | 4.28 |
| Cramps in leg, foot or bottom | 3.30 | 3.84 | 3.00 | 2.67 | 2.42 | 4.18 |
| Easily feel too cold | 3.29 | 5.00 | 4.70 | 3.97 | 2.44 | 4.14 |
| Feeling anxious for no reason | 3.28 | 4.06 | 3.45 | 3.67 | 2.20 | 4.34 |
| Jittery. easily startled, often worried | 3.25 | 4.22 | 3.53 | 3.60 | 2.08 | 4.42 |
| Itchy eyes | 3.24 | 3.80 | 3.10 | 2.93 | 2.32 | 4.16 |
| Fatigue increasing after a cold or sore throat | 3.21 | 3.97 | 4.05 | 2.47 | 2.22 | 4.26 |
| Headaches | 3.21 | 4.01 | 3.93 | 3.16 | 2.34 | 4.08 |
| Mental fog | 3.21 | 5.16 | 5.29 | 3.53 | 2.04 | 4.44 |
| Numbness/ tingling/ pins and needles | 3.20 | 4.55 | 3.76 | 2.78 | 2.18 | 4.22 |
| Thirsty all the time | 3.20 | 4.22 | 3.60 | 2.91 | 2.22 | 4.18 |
| Bloating of the stomach | 3.19 | 4.02 | 3.55 | 4.66 | 2.67 | 3.69 |
| Racing heart | 3.19 | 3.49 | 3.70 | 2.75 | 2.30 | 4.08 |
| Pain in legs and arms (which is not due to hard exercise) | 3.18 | 5.50 | 4.43 | 2.60 | 2.12 | 4.30 |
| Blocked nose | 3.17 | 3.47 | 3.10 | 2.85 | 2.76 | 3.59 |
| Pain increasing the day after you are active | 3.17 | 5.36 | 4.80 | 2.64 | 1.88 | 4.57 |
| Chest pain | 3.03 | 3.10 | 2.68 | 2.09 | 2.02 | 4.00 |
| Very vivid dreams | 3.03 | 3.56 | 3.59 | 3.14 | 2.14 | 3.94 |
| Depression | 3.02 | 3.82 | 3.07 | 3.02 | 2.02 | 4.00 |
| More clumsy than others | 2.94 | 4.21 | 4.27 | 2.75 | 1.62 | 4.31 |
| Running nose | 2.94 | 3.22 | 2.86 | 2.98 | 2.52 | 3.37 |
| Swollen painful joints | 2.91 | 4.14 | 2.93 | 2.25 | 1.98 | 3.86 |
| Face flushes | 2.87 | 3.54 | 2.82 | 2.52 | 1.88 | 3.86 |
| Heartburn | 2.74 | 3.22 | 2.64 | 3.14 | 2.27 | 3.23 |
| Pain moving from one place of body to another on different days | 2.68 | 5.18 | 3.88 | 2.25 | 1.55 | 3.85 |
| Ringing in ears | 2.66 | 3.34 | 3.20 | 2.58 | 2.10 | 3.22 |
| Intolerant to some food | 2.62 | 3.08 | 3.52 | 4.42 | 2.06 | 3.18 |
| Sensitivity to bright lights | 2.51 | 4.30 | 4.37 | 2.63 | 1.86 | 3.16 |
| Constipation | 2.49 | 3.51 | 2.90 | 3.43 | 1.80 | 3.18 |
| Feeling very ill for no reason | 2.48 | 3.53 | 4.38 | 2.32 | 1.34 | 3.65 |
| Stomach pain | 2.41 | 3.44 | 3.34 | 4.55 | 1.73 | 3.08 |
| Nausea for no reason | 2.40 | 3.21 | 3.57 | 3.09 | 1.82 | 3.00 |
| Head cold, sore throat or 'flu | 2.32 | 2.41 | 2.79 | 2.08 | 2.04 | 2.59 |
| Feeling faint | 2.21 | 2.72 | 3.53 | 2.32 | 1.54 | 2.90 |
| Sensitivity to noise | 2.17 | 4.66 | 4.53 | 2.89 | 1.53 | 2.80 |
| Skin rash | 2.17 | 2.16 | 2.08 | 1.93 | 1.84 | 2.51 |
| Diarrhoea | 2.16 | 2.78 | 2.62 | 4.02 | 1.70 | 2.62 |
| Nightmares/night terrors | 2.13 | 2.49 | 2.47 | 2.10 | 1.18 | 3.10 |
| Twitching of eyelid | 2.07 | 2.84 | 2.51 | 2.11 | 1.44 | 2.71 |
| Choking sensations | 2.06 | 2.05 | 1.72 | 1.57 | 1.32 | 2.80 |
| Double vision | 2.03 | 2.23 | 2.47 | 1.44 | 1.26 | 2.80 |
| Mouth ulcers (sores in mouth) | 2.01 | 2.14 | 2.02 | 1.84 | 1.50 | 2.53 |
| Boils or pimples on face or body | 1.93 | 2.38 | 2.60 | 2.14 | 1.32 | 2.55 |
| Twitching other than eyelid | 1.86 | 2.62 | 2.45 | 1.47 | 1.32 | 2.42 |
| Loss of voice | 1.83 | 1.77 | 1.86 | 1.33 | 1.41 | 2.24 |
